# Towards enhanced understanding of idiopathic ketotic hypoglycemia: a literature review and introduction of the patient organization, Ketotic Hypoglycemia International

**DOI:** 10.1186/s13023-021-01797-2

**Published:** 2021-04-13

**Authors:** Danielle Drachmann, Erica Hoffmann, Austin Carrigg, Beccie Davis-Yates, Valerie Weaver, Paul Thornton, David A. Weinstein, Jacob S. Petersen, Pratik Shah, Henrik Thybo Christesen

**Affiliations:** 1 Ketotic Hypoglycemia International (KHI), Skanderborg, Denmark; 2 School of Social Science, Nottingham Institute of Education, Nottingham, UK; 3 Cook Children’s Medical Center, Fort Worth, TX USA; 4 Glycogen Storage Disease Program, University of Connecticut, Farmington, CT USA; 5 Novo Nordisk A/S, Bagsværd, Denmark; 6 Endocrinology Department, The Royal London Children’s Hospital, Barts Health NHS Trust and Queen Mary University London, London, UK; 7 Department of Clinical Research, University of Southern Denmark, Odense, Denmark; 8 Hans Christian Andersen Children’s Hospital and Steno Diabetes Centre Odense, Odense University Hospital, JB Windsloews Vej 4, 5000 Odense C, Denmark

**Keywords:** Idiopathic ketotic hypoglycemia, Children, Glycogen storage disease, Rare disease, Ketone bodies, Hypoglycemia

## Abstract

**Background:**

Idiopathic Ketotic hypoglycemia (IKH) is a diagnosis of exclusion. Although considered as the most frequent cause of hypoglycemia in childhood, little progress has been made to advance the understanding of IKH since the medical term was coined in 1964. We aimed to review the literature on ketotic hypoglycemia (KH) and introduce a novel patient organization, Ketotic Hypoglycemia International (KHI).

**Results:**

IKH may be diagnosed after the exclusion of various metabolic and hormonal diseases with KH. Although often mild and self-limiting, more severe and long-lasting IKH occurs. We therefore divide IKH in physiological KH and pathological KH, the latter defined as recurrent symptomatic, or occasionally symptomatic, episodes with beta-hydroxybutyrate ≥ 1.0 mmol/L and blood glucose < 70 mg/dL (3.9 mol/L), in the absence of prolonged fasting, acute infections and chronic diseases known to cause KH. Pathological KH may represent undiscovered diseases, e.g. glycogen storage disease IXa, Silver–Russel syndrome, and ketone transporter defects, or suggested novel disease entities identified by exome sequencing. The management of KH aims to prevent hypoglycemia, fatty acid oxidation and protein deficiency by supplying adequate amounts of carbohydrates and protein, including nutritional therapy, uncooked cornstarch, and sometimes continuous tube feeding by night. Still, intravenous dextrose may be needed in acute KH episodes. Failure to acknowledge that IKH can be more than normal variation may lead to under-treatment. KHI is a non-profit, patient-centric, global organization established in 2020. The organization was created by adult IKH patients, patient family members, and volunteers. The mission of KHI is to enhance the understanding of IKH while advocating for patients, their families and the continued research into KH.

**Conclusion:**

IKH is a heterogeneous disorder including physiological KH and pathological KH. IKH may represent missed diagnoses or novel disease entities, but shares common management principles to prevent fatty acid oxygenation. KHI, a novel patient organization, aims to enhance the understanding of IKH by supporting IKH families and research into IKH.

## Introduction

Due to the lack of cerebral energy stores the brain depends on a continuous supply of glucose as a primary energy substrate. The brain is also capable of utilizing ketones, which occurs with fasting or chronic feeding of a high fat/low carbohydrate diet. The integration of metabolic pathways (glycogenolysis, gluconeogenesis, and fatty acid oxidation) and multiple counter-regulatory hormones (glucagon, epinephrine, growth hormone, and cortisol) combine to maintain normoglycemia. Hypoglycemia in children older than one month is uncommon, even in the setting of fasting. Ketosis during hypoglycemia, here referred to as ketotic hypoglycemia (KH), simply describes the physiologic changes (increased ketogenesis) which should occur in the setting of counter-regulation [1]. Despite more recent advances, only a minority of children with KH receive an endocrine or metabolic disorder diagnosis. The majority of KH patients are classically diagnosed as having "idiopathic" ketotic hypoglycemia (IKH), also known as accelerated starvation. IKH is considered the most frequent cause of hypoglycemia in childhood [2–4]. Data about the incidence and prevalence of KH and IKH are missing, but KH is the most common diagnosis applied to episodes of hypoglycemia in children in emergency departments in the US [2, 3]. While IKH is currently defined as a collective set of symptoms not related to an underlying pathology, severe IKH might still be considered as a rare disease entity in and of itself. A search of Orphanet for “idiopathic ketotic hypoglycemia” and “ketotic hypoglycemia” yielded neither an Orpha number, an Online Mendelian Inheritance in Man (OMIM) geno- or phenotype reference number, or an ICD-10 number. KH is referenced in the Human Phenotype Ontology (HPO) as HP:0012734, a sign of other known rare diseases [5, 6].

Little progress has been made to advance the understanding of IKH since 1964, where unexplained KH was reported in eight children after exclusion of hormonal and metabolic diseases [7]. IKH has been considered by some as a non-pathological condition in infants, representing the lower tail of the Gaussian normal distribution for fasting tolerance [8]. However, some children with unexplained KH, or IKH, are more severely affected and do not go into remission before school age and may even have IKH in adulthood. Such individuals had not had a patient organization with which to gain support, scientific information or research data, and currently do not have any standardized clinical management guidelines to follow. The aim of this paper is to review the literature on KH and introduce a novel patient organization, Ketotic Hypoglycemia International (KHI). Because much of the research available is centered on the pediatric population, this paper focuses solely on IKH in children.

## Metabolism of glucose, fats and ketones

In healthy individuals maintenance of stable plasma glucose (PG) concentration is easily performed by the body’s neuroendocrine and metabolic defenses, and potential consequences from prolonged fasting are low. However, a disproportionate balance between cellular glucose entry and glucose outflow secondary to deficient delivery of glucose into circulation, excessive glucose removal from circulation, or both, will place the body in a state of hypoglycemia [9, 10]. While glucose entry from the blood to the brain neurons and to liver cells is dependent on glucose concentration gradients, most other tissues (primarily muscle tissue) are dependent on insulin for glucose entry to the cells. As PG begins to fall, the pancreas stops producing insulin and decreases the amount of glucose available to most tissues, with the exception of the brain.

**Fig. 1:**
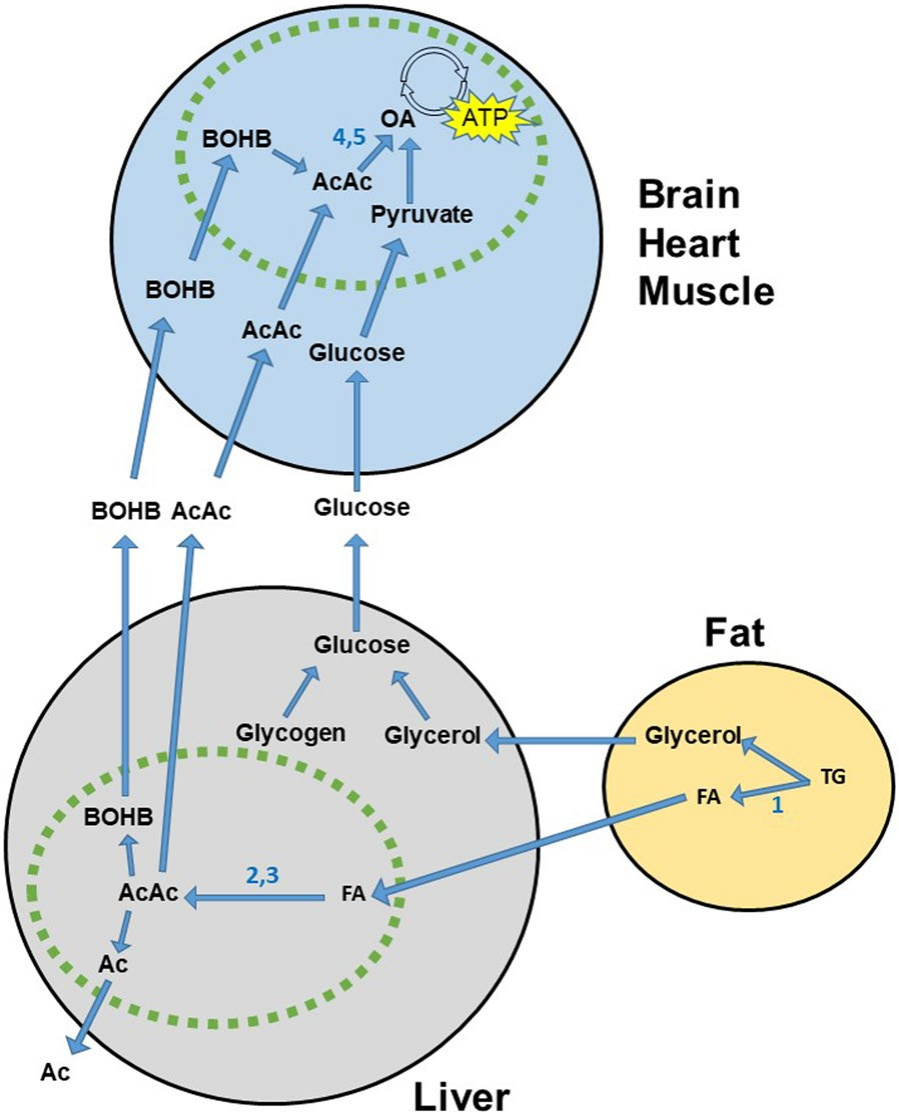
Selected details on the glucose and ketone body metabolism. Representation of selected details of the metabolism at the low insulin—high insulin counter-acting hormones state. Only cells of the liver, fat tissue and major fuel consuming organs are shown. Gluconeogenesis from amino acids, lactate/Cori cycle, and pathway details are omitted. Green line: Mitochondrial membrane. Blue circle: Citric acid cycle. Key enzymes in ketogenesis (arrows): (1) triglycerate lipase; (2) acetyl CoA carboxylase; (3) HMG CoA synthase. Key enzymes in ketolysis: (4) CoA-oxoacid transferase (SCOT); (5) michochondrial acetoacetyl CoA thiolase (MAT; beta-ketothiolase). *Ac* acetone, *AcAc* acetoacetate, *ATP* adenosine triphosphate, *BOHB* beta-hydroxybutyrate, *FA* fatty acids, *OA* oxaloacetate, *TG* triglyceride

If the PG continues to fall, the counter-regulatory hormones glucagon, cortisol, growth hormone, epinephrine (adrenaline) and norepinephrine (noradrenaline) are released into circulation, and the breakdown of triglycerides to glycerol and fatty acids in the adipocytes (lipolysis) occurs, Fig. 1 [10–12]. The glycerol is then converted to glucose in the liver (gluconeogenesis). Both glycerol and amino acids serve as substrates for glucose production in the hormonally controlled gluconeogenesis. The free fatty acids from lipolysis are processed by liver cell mitochondria into acetoacetate (AcAc), beta-hydroxybutyrate (BOHB), and, to a lesser degree, acetone, which together are named ketone bodies. These ketone bodies are an alternative source of energy for the brain and muscles when glucose is unavailable [13, 14].

Fuel production in infants and children is similar to that of adults; however, due to their larger brain size in proportion to their body weight, PG concentrations in infants and children drop more rapidly and elevated ketone body levels (hyperketonemia) develop sooner [10, 13]. The brain has back-up stores of fuel in the form of glycogen to compensate for low PG levels [14–16]. These glycogen stores only last a few minutes, so individuals that cannot regulate their PG levels are at risk for interruption of glucose delivery to the brain, putting them in danger of devastating consequences. Recovery is usually complete from short-lived episodes of hypoglycemia, though prolonged and severe hypoglycemia can cause permanent brain damage [14, 17–19].

## Ketosis

Ketone bodies can protect the brain from damage by providing an alternate fuel source. Ketones provide 2–6% of the body’s energy requirements after an overnight fast and up to two-thirds of the brain’s energy needs after a prolonged fast or during starvation [13, 14]. Normal infants and children can also develop hyperketonemia during illness, after bouts of vomiting or diarrhea, or after prolonged fast with an inverse relation to age [20–24].

The normal serum concentration of ketones measured as 3-BOHB is assay-dependent, but invariably given as < 0.6 mmol/L [25, 26]. Hyperketonemia is most often defined as BOHB concentrations between 1.0 and 3.0 mmol/L [25]. This definition reflects to the authors’ clinical experience the threshold of symptomatic ketosis, which begins around 1.0 mmol/L.

Ketoacidosis is defined as BOHB > 3.0 mmol/L with resultant metabolic acidosis [25, 26]. The metabolic acidosis occurs as the rapid rise in ketone bodies and subsequent saturation of skeletal muscle uptake mechanisms causes an increase in serum hydrogen ion concentration that overwhelms the body’s natural buffering system [25]. In studies with prolonged fasting in healthy controls, the 90^th^ percentile of BOHB reached 1.0 mmol/L after 15 h fast in 1–12 months old infants; 0.9 mmol/L at age 1–7 year; and 0.3 mmol/L at age 7–15 years [22]. The 97.5^th^ percentile of BOHB after 14 h fast was 1.5 mmol/L in 3-y-olds; 0.7 mmol/L at age 9 years; and after 40 h fast 8.9 mmol/L at age 6–11 years [23, 24].

In the acute phase, high ketones can cause nausea, vomiting, abdominal pain, muscle cramps, headache, lethargy, and at its most severe, coma and polypnea (Kussmaul breathing) [3, 26]. Acetone excretion by the lungs results in a characteristic aromatic smell on the breath. If measures are not taken to arrest ketogenesis and flush out excess ketones from the body, ketoacidosis can develop, constituting a need for emergent intervention [25]. Chronic ketosis can result in poor growth, hepatic transaminase elevation, and osteoporosis [27].

## Diagnosis of ketotic hypoglycemia

### Definition of KH and IKH

IKH has long been considered the lower end of normal variation for fasting tolerance in infants [8] However, IKH seems to represent a broad clinical spectrum from occasional mild KH in infancy to severe and more long-lasting KH, sometimes with other clinical or biochemical manifestations. We propose that IKH be split into physiological KH and pathological KH. We define pathological KH as recurrent symptomatic, or occasionally symptomatic, episodes with BOHB ≥ 1.0 mmol/L and blood glucose < 70 mg/dL (3.9 mmol/L), in the absence of prolonged fasting, acute infections and chronic diseases known to cause KH. This definition allows for episodes with euglycemic hyperketosis.

Physiological KH is most commonly seen in normal children with intercurrent acute illness causing prolonged starvation and increased metabolism due to fever. Moreover, KH may be frequent in conditions with excess starving or malnutrition. In a few studies, KH was reportedly common in African children with chronic malnutrition or severe malaria [28, 29].

Pathological forms of KH are seen in children with deficiencies in the counter regulatory hormones such as cortisol or growth hormone, or in metabolic conditions such as glycogen storage disease (GSD, specifically GSD 0, III, VI, and IX), disorders of ketone transport and ketone utilization, and defects in protein metabolism, Table 1 [30, 31]. Specific syndromes with KH should be identified, e.g. Silver–Russell syndrome where more than one in four have KH after a simple overnight fast [32, 33].

**Table 1:** Causes to ketotic hypoglycemia in children

	Gene or chromosome	Inheritance
Hormonal		
Growth hormone deficiency, or resistance	Genetic or acquired	Variable
ACTH deficiency or resistance; cortisol deficiency	Genetic or acquired	Variable
Glucagon deficiency*	*GCG, DBH*	N/D
Dopamine beta-hydroxylase deficiency*	*GCG, DBH*	N/D
Metabolic		
Glycogen storage disease (GSD)		
GSD 0; glycogen synthase deficiency	*GYS2*	AR
GSD III; glycogen debranching enzyme deficiency	*AGL*	AR
GSD VI; glycogen phosphorylase deficiency	*PYGL*	AR
GSD IX; phosphorylase kinase subunit deficiencies	*PHKA2, PHKB, PHKG2*	X-linked, AR
Glucose metabolism and transport		
Phosphoglucomutase I deficiency	*PMG1*	AR
Pyruvate carboxylase deficiency	*PC*	AR
Organic acidemias		
Maple syrup urine disease, propionic aciduria, methylmalonic aciduria	Multiple genes	AR
Ketone body transport and metabolism		
Monocarboxylase transporter 1 defect	*SLC16A1 (MCT1)*	AR,AD
Ketolysis		
Succinyl CoA oxoacid transferase deficiency	*SCOT*	AR
Mitochondrial acetoacetyl-CoA thiolase (beta-ketothiolase) deficiency	*ACAT1*	AR
Syndromes		
Silver–Russel syndrome	11p15 or 7**	Mostly sporadic
Prader–Willi syndrome	15q11-q13***	Mostly sporadic
Fanconi–Bickel syndrome	*SLC2A2 (GLUT2)*	AR
Secondary KH to chronic malnutrition, severe malaria, other chronic diseases	-	-
Idiopathic ketotic hypoglycemia		
Physiological KH in prolonged fasting or acute illness	-	-
Pathological KH	-	-
IGF2BP1 deficiency*	*IGF2BP1*	N/D
Sodium glucose co-transporter 2 defect*	*SLC5A2*	N/D
PEP carboxykinase 1 and G-6P catalytic transcriptional induction*	*NCOR1*	N/D
Mitosis gene A-related kinase 11 defect*	*NEK11*	N/D

*AD* autosomal dominant, *AR* autosomal recessive, *N/D* no data, *PEP* phosphoenolpyruvate, *G-6P* glucose 6-phosphate. The list is not fully inclusive

*Suggested, not well-established causes to KH

**Several mechanisms, rare other mechanisms, or unknown

***Paternal deletion, maternal uniparental disomy, or imprinting defect

IKH may be diagnosed when all the above causes have been excluded. Because there may be many yet undiscovered specific etiologies for IKH, it can be considered as a condition with disturbed glucose homeostasis, the cause being failure of meaningful hepatic gluconeogenesis rather than an accelerated glucose oxidation rate [9, 31, 34]. In keeping, hypoalaninemia has been found in children with KH compared to age-matched controls [35]. The body's inability for self-regulation results in a state of low PG levels with elevated ketones and various outward signs and symptoms shown by the child.

No uniform diagnostic criteria for PG or ketone body concentrations have been established for the diagnosis of KH. Hypoglycemia in adults and children cannot be defined by a single PG cut-off [13, 36]. van Veen et al. found that children below 2 years had physiological KH with PG below 70 mg/dL (3.9 mmol/L) and hyperketonemia after fasting for 15 h or shorter [21]. This study, however, included children with potential pathological KH.

In children with the diagnosis of IKH, hyperketonemia with euglycemia appears to occur more frequent than hyperketonemia with hypoglycemia [26]. In the author’s experience (HTC), this may, however, be the result of non-recorded nocturnal hypoglycemia as demonstrated with continuous glucose monitoring (CGM).

The demonstration of Whipple’s triad for hypoglycemia (symptoms and signs, low PG and relief of symptoms and signs after normalization of PG) is often used to support the diagnosis of hypoglycemia, but is not straight forward in KH. Symptoms are difficult to read in infants and recurrent hypoglycemia leads to hypoglycemia-associated autonomic failure with blunting of the autonomic symptoms of hypoglycemia, which results in hypoglycemia unawareness, irrespective of age [37, 38]. The clinical picture of IKH is described below.

### Clinical picture of IKH

Episodes of KH are usually seen from around 6 months’ age, with the hypoglycemia attributed to poor oral intake or illness [3, 9, 39]. In a series of 62 children with KH, 29% had, however, no history of a precipitating event that could explain the hypoglycemia [40]. To the authors’ experience, increased physical activity and poor food intake the day before can be identified in a higher number of patients after detailed evaluation at the time of a KH event.

Remission of IKH is classically thought to occur between 6–7 years of age when children have increased their muscle mass and decreased their glucose requirement per unit of body mass [40, 41]. In some patients, episodes of KH may persist past puberty into adulthood, and family investigations may reveal parents with a history of KH [42]. This should prompt further diagnostic investigations and be regarded as pathological KH.

Children with KH may exhibit a wide range of symptoms and signs. Younger children cannot be relied upon to accurately recognize and communicate symptoms of hypoglycemia [13, 37, 38, 43–45]. It is often up to the primary caregiver(s) to interpret whether the child is experiencing a hypoglycemic event and if intervention is warranted. Episodes frequently occur in the morning after an overnight fast (10–12 h). In response to the hypoglycemic state overnight, ketone development has already begun, making management of symptoms a challenge for caregivers [40].

Symptoms and signs of the combination of hyperketosis and hypoglycemia in KH include nausea, abdominal pain, vomiting, headache, lethargy, pallor, sweating, tremors, muscle cramps, visual/sensory disturbances, speech difficulty, mental confusion, bizarre behavior (such as crying for no reason), temper tantrums, restlessness, personality changes, seizures and coma [3, 7, 14, 26, 40, 41]. The presence of neuroglycopenic symptoms despite the brain-protective hyperketonemia may suggest a ketone utilization or ketone transport defect in these patients. What makes KH particularly complex is that some of the symptoms, such as muscle cramps, headache and abdominal pain, may result from either hypoglycemia or hyperketonemia.

Some children with IKH are small and thin for their age, with minimal fat deposits or muscle development [41]. Such children have little to no tolerance for fasting and often display poor eating habits. The poor appetite may be caused by hyperketonemia, inducing a vicious cycle provoking further KH.

### Diagnostic procedures in IKH

If the KH is not investigated during a spontaneous acute episode with severe symptoms and signs, a controlled fasting test in the hospital setting can help to establish the KH diagnosis and to give an impression of its severity. Dependent on age, an overnight fasting study can be performed until the glucose is either < 50 mg/dl (2.8 mmol/L) or the BOHB is > 2 mmol/L. At the end of the fasting study, a diagnostic critical sample is obtained. A controlled fasting study with frequent monitoring of both glucose and BOHB allows the physician to identify when the BOHB rises above 1 mmol/L and thus gives the family an understanding of the child’s fasting tolerance. Permanent or excessive ketosis should lead to investigations for ketone transporter or utilization defects.

Once the diagnosis of IKH is established, the frequency and severity of KH should be determined as part of the initial work up. Home monitoring with point of care (POC) meters with testing for glucose and ketones should be performed, particularly on waking in the morning or with symptoms until a true pattern emerges. CGM may be helpful, but are not rigorously studied in IKH and cannot stand alone due to inaccuracy at lower glucose levels. The episodical nature of KH may call for prolonged home monitoring in pathological KH (see also the Management section below).

Patients with pathological KH represent a diagnostic challenge to physicians and caretakers. Pathological KH with or without atypical manifestations should lead to expanded diagnostic procedures. A clinical example is given in Table 2.

**Table 2:** A child with idiopathic ketotic hypoglycemia

A white Danish boy, born at term, birth weight 3425 g, uncomplicated. No siblings. The parents’ history was without symptoms of hypoglycemia or diabetes. Since 4 months’ age, the boy had recurrent vomiting and poor appetite. By age 4 years, poor appetite with recurrent headache, dizziness and loss of consciousness in the morning led to investigations for hypoglycemia. Morning bedside glucose were down to 3.2 mmol/L with blood ketones up to 2.6 mmol/L with repeat KH in the following years. Height and weight were normal. From 8 years, his KH aggravated with glucose down to 2.0 mmol/L, ketones up to 5.6 mmol/L and attacks both in the morning and in the daytime	
Normal findings included growth, hematology, electrolytes, liver and kidney counts; IGF1 and IGF-BP3, thyroid hormones, fasting insulin, cortisol response to hypoglycemia, synacthen test cortisol response; urine metabolic screening (except for ketone bodies), plasma amino acids, very long-chained fatty acids, other peroxisomal enxymes and biotinidase; abdominal ultrasound; a 29-gene NGS panel for glycogen storage diseases; skin biopsy fibroblast culture for metabolic enzyme concentrations; and muscle biopsy analyses (histology, electron microscopy, respiratory chain enzymes and pyruvate dehydrogenase activity, mtDNA for larger deletions and missense mutations, lysosomal enzymes group 1). An i.m. glucagon test (0.03 mg/kg) showed a glucose response from 4.3 to 5.4 mmol/L without rebound hypoglycemia or hyperlactatemia. Plasma lactate was repeatedly normal, while plasma pyruvate was mildly elevated on three repeat occasions, 126–138 (ref. 34–80) µmol/L	
The boy was monitored with bedside glucometer with ketone sticks and continuous glucose monitoring. Treatment included complex carbohydrates, proteins, snacks between meals, uncooked corn starch and overnight maltose infusion by gastric tube until last follow-up aged 11 years	

### Search of new causes to KH

A predominance of IKH in males has been reported [3, 40]. This may suggest an unrecognized disease on the X-chromosome such as GSD IXa in these children. Indeed, several patients diagnosed with KH or IKH have been found to have mutations in *PHKA2* [30, 40, 46]. By use of trio exome sequencing, research has identified mutations not only in *PHKA2*, but also in SLC16A1 *(MCT1)*, *NCOR1, IGF2BP1, SGLT2* and *NEK11* as (potential) novel causes of more atypical children with otherwise unexplained KH [42, 47].

*SLC16A1* codes for monocarboxylase transporter 1 (MCT1), which mediates transport of pyruvate, lactate and ketone bodies across cell membranes. Patients with heterozygous or homozygous inhibiting mutations in *SLC16A1* presented with moderate or profound ketosis and sometimes hypoglycemia at fasting or during infections, with onset in the first years of life [40, 47, 48]. In some of the patients, migraine, exercise intolerance, developmental delay, microcephaly and abnormal MRI of the brain were noted.

The study of Alhaidan et al. identified predicted disease-causing heterozygous DNA variants in *NCOR1, IGF2BP1, SLC5A2* (*SGLT2*) and *NEK11,* respectively, in four children with otherwise unexplained KH [42]. The NCOR1/HDAC3 complex is involved in the regulation of liver phosphoenolpyruvate carboxykinase 1 (PCK1), glucose 6-phosphatase catalytic (G6PC) and hepcidin. The KH patient with an *NCOR1* mutation had loose stools and iron deficiency anemia as additional features, the latter possibly caused by hepcidin overexpression. Of note, *G6PC* homozygous mutations leads to GSD Ia with non-ketotic hypoglycemia, hepatomegaly and iron deficiency [49].

*IGF2BP1* (not to be confused with *IGFBP1*) is a sparsely described translational regulator of several proteins, including IGF2, which is involved in cell growth and differentiation and activates the insulin receptor. Patients with IGF2-producing tumors have impaired glycogenolysis and gluconeogenesis with hypoglycemia and suppressed insulin secretion [50–52]. The KH patient with a heterozygous *IGFBP1* mutation had short stature and retarded bone age without identifiable defects in the growth hormone axis as additional features.

Heterozygous mutations in *SLC5A2*, which encodes the sodium glucose co-transporter 2 (SGLT2), cause familial renal glucosuria (FRG) [53]. The KH patient with a heterozygous *SLC5A2* mutation had intermittent glucosuria, rapid clearance of glucose and drastically increased fasting p-GLP1 as additional features, suggesting KH in infancy as a novel manifestation of FRG.

The mitosis gene A-related kinase 11 gene *NEK2* has not been linked to other diseases in humans than KH. In a heterozygous *NEK11* mutation mouse model, a significant reduction in glucose levels was the only reported feature [54]. The KH patient with a heterozygous *NEK11* mutation had recurrent migraine, cognitive and motor deficits, mild liver affection, absent glucose response to i.m. glucagon and decreased p-IGFBP3 as additional features.

A number of children with pathological KH remains unexplained despite trio exome sequencing [42]. Pathological KH may well represent a disease entity with several different unidentified monogenetic, digenic, polygenetic, or epigenetic causes [12, 30]. A case report with KH in one, but not the other homozygotic twin illustrates the complexity of KH [55].

## Management of idiopathic ketotic hypoglycemia

The monitoring and treatment of children with IKH should be individualized according to severity, frequency and response. While physiological KH only will need attention in case of acute illness or prolonged fasting, pathological KH may need everyday monitoring and treatment. Common management principles for pathological KH are presented in Table 3. POC glucometers have different diagnostic performances and are less reliable in the lower range [56–58]. Moreover, finger prick capillary PG concentrations derive from the optimal venous PG concentration, and the automatic 1.1 conversion factor from whole blood POC glucometers to report PG concentrations may not reflect the actual erythrocyte volume fraction of an infant. Despite these limitations, home monitoring with POC testing for PG and ketones should be used in monitoring KH in its severe forms. POCT for blood BOHB should be preferred from the less precise urine stix AcAc [59].

**Table 3:** Management of pathological KH in children

	Range of use
Monitoring	
Bedside glucometer and blood ketones	Initial work up
	Fever, vomiting or diarrhea; at symptoms/ morning fasting values/ frequent daily
Continuous glucose monitoring*	Initial work up; for months or years in more severe, pathological KH
Prevention	
Dietary	
Complex carbohydrates, protein	Before sleep only; for every meal
Meal interval	Dependent on age, severity and frequency of KH
Uncooked corn starch	½–1 (− 2) g/kg, 1–4 × daily
Long-release corn starch	Severe, frequent KH
Continuous gastrostomy tube feeding	Severe, frequent KH. Night feeding with maltose 1/2–1 g/kg/h; other tube feeding products
Acute treatment	
Dietary	
Sugar-rich drinks and food	KH attacks without compromised swallowing. Add complex carbohydrates, eventual protein
Buccal carbohydrate gel application	KH attacks with compromised swallowing. 1/2–1 tube, eventually repeated
Medication	
I.m. glucagon	Severe KH attacks with unconsciousness. 30–40 mcg/kg, maximal 1 mg. Only if proven efficient and safe at specialist center
I.v. glucose or dextrose	Severe KH attack. Ensure PG > 3.9 mmol/L (70 mg/dL). Continue until blood ketones < 0.5 mmol/L

*KH* ketotic hypoglycemia, *PG* plasma glucose

*Continuous glucose monitoring cannot stand alone due to inaccuracy at low glucose concentrations, and needs further research specifically in KH

As the frequency and severity of KH episodes vary, the need for home monitoring must be individualized for each patient. Severely affected children may benefit from CGM, although low glucose values or decreasing trend alarms should be controlled with a glucometer to improve precision.. As hyperketonemia may be of clinical significance without simultaneous hypoglycemia, blood ketone monitoring may be recommended for normoglycemic patients exhibiting signs and symptoms of illness.

POC monitoring may be an economic burden on the family, as well as a logistical challenge for custodial daycare and school personnel. On the other hand, POC testing may facilitate the correct identification of episodic behavioral changes as symptoms of KH instead of general “bad behavior”, psychosocial reactions, or ADHD, which had occurred according to the anecdotal experience in the new patient organization, KHI. Still, the need of monitoring should be evaluated regularly to avoid both “over”- and “under”-monitoring.

The Pediatric Endocrine Society recommends maintaining a PG concentration of > 70 mg/dL (3.9 mmol/L) as goal of treatment for hypoglycemia in infants and children, however allowing for an individualized, tailored approach to management [13]. To the authors´ experience, children with pathological KH benefit from an individualized PG goal to keep BOHB < 1.0 mmol/L. This may fit with a PG kept > 70 mg/dL (3.9 mmol/L), but an individual PG goal for each child to avoid hyperketosis should be determined in the diagnostic process.

Nutritional therapy, avoidance of prolonged fasting, increased frequency of feedings, and close monitoring of oral intake, especially in times of stress or high activity, are suggested to mitigate episodes of hypoglycemia [12, 13]. Uncooked cornstarch has been used to extend the times between feedings by delaying onset of hypoglycemia. Traditional cornstarch has been used for 40 years to prevent nocturnal hypoglycemia, with the measured length of time for digestion being 3–4 h [27, 60].

For some children with pathological KH, as in those with KH due to GSD I, an overnight administration of cornstarch is necessary. In addition to cornstarch therapy, children with pathological KH that are unable to maintain fasting for any significant length of time may be evaluated for gastrostomy tubes for bolus or continuous tube feedings [27, 60]. Protein-rich diet or dietary products should be considered if low protein or prealbumin concentrations are documented. Proteins provide substrate for gluconeogenesis and may reduce muscle cramps associated to KH.

Nausea and vomiting caused by ketones may lead to further decline in PG if hyperketosis is not recognized and treated. The acute treatment principle includes administration of high glycemic index (i.e. dextrose-rich) foods or drinks to provide energy from glucose metabolism instead of fatty acid metabolism which leads to further ketone body formation.

If hypoglycemia is unable to be corrected or the child becomes increasingly unresponsive, emergency intervention should be sought. Buccal carbohydrate gel administration, intravenous (IV) dextrose or intramuscular glucagon (if proven effective and safe by formal testing) may be administered to raise PG concentrations to ≥ 70 mg/dL (3.9 mmol/L). Of note, some patients with IKH have a normal i.m. glucagon response without provoking lactate acidosis, unlike the response in those with GSD I [3, 35, 39].

Elimination of ketones can take hours, and if the child is in an acidotic state, IV fluid therapy may be needed to return the patient to homeostasis. Intravenous dextrose can help to stop ketogenesis and lipolysis, and rehydration assists in renal excretion of ketones [12, 25, 41]. A better understanding of KH through identification of novel genetic causes may help physicians to target the treatment of children with pathological KH.

## Ketotic Hypoglycemia International

Established in January, 2020, Ketotic Hypoglycemia International (KHI) is a new, worldwide patient organization for families affected by idiopathic ketotic hypoglycemia (IKH). Our scientific advisory board and organization board of directors are volunteers. The mission of KHI is to enhance the understanding of IKH for the benefit of children, parents, and families who have been affected by IKH. KHI aims to support patients and their families by sharing knowledge about IKH, and to support the continued research into etiology, monitoring, and treatment.

We define our patient target group as patients with KH without a known clinical metabolic or hormonal etiology. This is to differentiate KHI from patient organizations for GSD, growth hormone deficiency and adrenal insufficiency, even though members of these groups may also experience KH symptoms. KHI welcomes all patients who suffer from KH in our support group, where we will assist them in connecting with other disease specific support organizations.

### Which challenges are we facing?

Current literature provides a broad, unclear etiology and pathophysiology of IKH, thereby limiting the scope of understanding especially the more severely affected patients. The KHI support group has the impression that pathological KH may often be underdiagnosed and inappropriately treated. For those with pathological KH there is the lack of support for both the patients and their primary caregivers. Rare diseases often come with additional stress to the family unit, as advocacy, research, and support falls on the patient and family members. It has been shown that in the case of a rare disease diagnosis, oftentimes the parent or caregiver will have a better understanding of the disease process than the healthcare team providing care for the patient. Limited access to services and government support, fragmented communication, and minimal experts in the field result in the need for family to step in as a patient medical navigator and advocate [61].

Advocating for a child without a diagnosis can become overwhelming to the family as social support dwindles and health care management becomes convoluted. The initial screening for KH is not standardized in many institutions, and when a diagnosis of IKH is made, parents have reported feelings that providers had offered minimal education and instruction on management. This results in families navigating uncharted territory with episodes of KH, hospitalizations, pushback from schools regarding symptom management and prevention plans, etc., while being explained that KH does not meet the definitions of a rare disease and therefore may not qualify for extra services.

KHI recognizes that pathological KH, as with many health conditions, is not only affecting the child, but creating daily challenges for the families and caregivers. The most significant of these challenges is the fear of hypoglycemic episodes that can lead to a range of complications and can have devastating consequences.

### What do we do?

KHI aims to globally unite leading hypoglycemia experts with patients, caregivers, and healthcare providers, and to establish a greater understanding of IKH in the hopes to facilitate improved treatment options and greater quality of life for individuals suffering from KH.

KHI was launched on a variety of social media platforms in January 2020 in an effort to connect with families from across the globe. The interest from families affected by KH was significant. We found that the majority of the families were affected by IKH and had been left in limbo with no recognized specialists to reach and no patient organization. The Facebook page for KHI, https://www.facebook.com/ketotichypoglycemiainternational/, has been exceptionally successful, leading to the formation of a Facebook group with more than 900 families thus far.

This is the first time these families have been able to enter into a support network of like-minded peers to share the daily difficulties presented with this condition, as well as offer and receive empathy and advice. We are committed to support and advocate for those in the KH community through our international KHI Parents Support Group by utilizing the contributions of general medical guidance made by our scientific advisory board, supporting research in the general well-being of KH-families, and furthering research into KH etiology, diagnosis, and management.

The organization aims to challenge current perceptions and attitudes to expand the recognition that IKH is more than a normal variation, and establish an international platform for future research collaboration. KHI strives to prevent IKH families from feeling silenced and underserved, as seen in other rare disease families [62]. KHI continues to bridge the gaps between doctors, families, providers, research, and awareness.

KHI strives to be recognized not only as a symptom-union, but as a rare disease partner, supporting research towards better understanding of the multifactorial causes of IKH and the genetic complexities of the numerous rare diseases it is associated with. All patients with KH share many of the same challenges, which is why collaboration with other rare disease patient organizations is of high priority.

## Conclusion

IKH is a clinically heterogeneous disorder including physiological KH and pathological KH. Pathological KH affects the health and well-being of its patients and their families. IKH patients currently do not have known causative genetic mutations or metabolic disorders, but novel causes may emerge in future studies. In addition to unknown etiology, patients with pathological KH have limited access to support, family resources, and medical management recommendations. KHI was formed as a novel patient organization to enhance the understanding of IKH, attempting to bridge the gap between patients, families, providers, researchers, and support, ultimately to improve the management of IKH and thereby to increase the quality of life for patients living with KH.

## Data Availability

Not applicable.

## References

[1] Owen OE, Reichard GA, Patel MS, Boden G (1979). Energy metabolism in feasting and fasting. Adv Exp Med Biol.

[2] White K, Truong L, Aaron K, Mushtaq N, Thornton PS (2020). The incidence and etiology of previously undiagnosed hypoglycemic disorders in the emergency department. Pediatr Emerg Care.

[3] Daly LP, Osterhoudt KC, Weinzimer SA (2003). Presenting features of idiopathic ketotic hypoglycemia. J Emerg Med.

[4] Pershad J, Monroe K, Atchison J (1998). Childhood hypoglycemia in an urban emergency department: epidemiology and a diagnostic approach to the problem. Pediatr Emerg Care.

[5] Köhler S, Carmody L, Vasilevsky N, Jacobsen JOB, Danis D, Gourdine JP (2019). Expansion of the Human Phenotype Ontology (HPO) knowledge base and resources. Nucleic Acids Res.

[6] Shefchek KA, Harris NL, Gargano M, Matentzoglu N, Unni D, Brush M (2020). The Monarch Initiative in 2019: an integrative data and analytic platform connecting phenotypes to genotypes across species. Nucleic Acids Res.

[7] Colle E, Ulstrom RA (1964). Ketotic hypoglycemia. J Pediatr.

[8] Senior B (1973). Ketotic hypoglycemia. A tale (tail) of Gauss?. J Pediatr.

[9] Bodamer OA, Hussein K, Morris AA, Langhans CD, Rating D, Mayatepek E (2006). Glucose and leucine kinetics in idiopathic ketotic hypoglycaemia. Arch Dis Child.

[10] Sprague JE, Arbeláez AM (2011). Glucose counterregulatory responses to hypoglycemia. Pediatr Endocrinol Rev.

[11] Gandhi K (2017). Approach to hypoglycemia in infants and children. Transl Pediatr.

[12] Ghosh A, Banerjee I, Morris AAM (2016). Recognition, assessment and management of hypoglycaemia in childhood. Arch Dis Child.

[13] Thornton PS, Stanley CA, De Leon DD, Harris D, Haymond MW, Hussain K (2015). Recommendations from the pediatric endocrine society for evaluation and management of persistent hypoglycemia in neonates, infants, and children. J Pediatr.

[14] Langdon DR, Stanley CA, Sperling MA, Sperling MA (2014). Hypoglycemia in the toddler and child. Pediatric endocrinology.

[15] Brown AM, Ransom BR (2007). Astrocyte glycogen and brain energy metabolism. Glia.

[16] Rich LR, Harris W, Brown AM (2019). The role of brain glycogen in supporting physiological function. Front Neurosci.

[17] Lucas A, Morley R, Cole TJ (1988). Adverse neurodevelopmental outcome of moderate neonatal hypoglycaemia. BMJ.

[18] Menni F, de Lonlay P, Sevin C, Touati G, Peigné C, Barbier V (2001). Neurologic outcomes of 90 neonates and infants with persistent hyperinsulinemic hypoglycemia. Pediatrics.

[19] Helleskov A, Melikyan M, Globa E, Shcherderkina I, Poertner F, Larsen AM (2017). Both low blood glucose and insufficient treatment confer risk of neurodevelopmental impairment in congenital hyperinsulinism: a multinational cohort study. Front Endocrinol.

[20] Saudubray JM, Marsac C, Limal JM, Dumurgier E, Charpentier C, Ogier H (1981). Variation in plasma ketone bodies during a 24-hour fast in normal and in hypoglycemic children: relationship to age. J Pediatr.

[21] van Veen MR, van Hasselt PM, de Sain-van der Velden MG, Verhoeven N, Hofstede FC, de Koning TJ (2011). Metabolic profiles in children during fasting. Pediatrics.

[22] Bonnefont JP, Specola NB, Vassault A, Lombes A, Ogier H, de Klerk JB (1990). The fasting test in paediatrics: application to the diagnosis of pathological hypo- and hyperketotic states. Eur J Pediatr.

[23] Lamers KJ, Doesburg WH, Gabreëls FJ, Romsom AC, Renier WO, Wevers RA (1985). Reference values of blood components related to fuel metabolism in children after an overnight fast. Clin Chim Acta.

[24] Lamers KJ, Doesburg WH, Gabreëls FJ, Lemmens WA, Romsom AC, Wevers RA (1985). The concentration of blood components related to fuel metabolism during prolonged fasting in children. Clin Chim Acta.

[25] Laffel L (1999). Ketone bodies: a review of physiology, pathophysiology and application of monitoring to diabetes. Diabetes Metab Res Rev.

[26] Millar R, Harding A (2019). Review article: accelerated starvation of childhood: have i judged ketones?. Emerg Med Australas.

[27] Weinstein DA, Steuerwald U, De Souza CFM, Derks TGJ (2018). Inborn errors of metabolism with hypoglycemia: glycogen storage diseases and inherited disorders of gluconeogenesis. Pediatr Clin N Am.

[28] Monde AA, Djessou SP, Camara CM, Tiahou GG, Koffi G, Djohan F (2010). Prevalence of ketotic hypoglycaemia among schoolchildren in the village of Ahoué in Côte-d'Ivoire. Bull Soc Pathol Exot.

[29] White NJ, Miller KD, Marsh K, Berry CD, Turner RC, Williamson DH (1987). Hypoglycaemia in African children with severe malaria. Lancet.

[30] Brown LM, Corrado MM, van der Ende RM, Derks TG, Chen MA, Siegel S (2015). Evaluation of glycogen storage disease as a cause of ketotic hypoglycemia in children. J Inherit Metab Dis.

[31] Dahlquist G, Gentz J, Hagenfeldt L, Larsson A, Löw H, Persson B (1979). Ketotic hypoglycemia of childhood—a clinical trial of several unifying etiological hypotheses. Acta Paediatr Scand.

[32] Wakeling EL, Brioude F, Lokulo-Sodipe O, O'Connell SM, Salem J, Bliek J (2017). Diagnosis and management of Silver–Russell syndrome: first international consensus statement. Nat Rev Endocrinol.

[33] Azcona C, Stanhope R (2005). Hypoglycaemia and Russell-Silver syndrome. J Pediatr Endocrinol Metab.

[34] Huidekoper HH, Duran M, Turkenburg M, Ackermans MT, Sauerwein HP, Wijburg FA (2008). Fasting adaptation in idiopathic ketotic hypoglycemia: a mismatch between glucose production and demand. Eur J Pediatr.

[35] Pagliara AS, Kari IE, De Vivo DC, Feigin RD, Kipnis DM (1972). Hypoalaninemia: a concomitant of ketotic hypoglycemia. J Clin Investig.

[36] Cryer PE, Axelrod L, Grossman AB, Heller SR, Montori VM, Seaquist ER (2009). Evaluation and management of adult hypoglycemic disorders: an Endocrine Society Clinical Practice Guideline. J Clin Endocrinol Metab.

[37] Cryer PE (1999). Symptoms of hypoglycemia, thresholds for their occurrence, and hypoglycemia unawareness. Endocrinol Metab Clin North Am.

[38] Hussain K, Bryan J, Christesen HT, Brusgaard K, Aguilar-Bryan L (2005). Serum glucagon counterregulatory hormonal response to hypoglycemia is blunted in congenital hyperinsulinism. Diabetes.

[39] Haymond MW, Pagliara AS (1983). Ketotic hypoglycaemia. Clin Endocrinol Metab.

[40] Kaplowitz P, Sekizkardes H (2019). Clinical and laboratory characteristics and follow up of 62 cases of ketotic hypoglycemia: a retrospective study. Int J Pediatr Endocrinol.

[41] Mridha A, Martin A (2019). Ketotic hypoglycemia in children: a review. Bangladesh J Child Health.

[42] Alhaidan Y, Larsen MJ, Schou AJ, Stenlid MH, Al Balwi MA, Christesen HT (2020). Exome sequencing revealed DNA variants in NCOR1, IGF2BP1, SGLT2 and NEK11 as potential novel causes of ketotic hypoglycemia in children. Sci Rep.

[43] Cryer PE (2013). Hypoglycemia-associated autonomic failure in diabetes. Handb Clin Neurol.

[44] Christesen HB, Brusgaard K, Beck Nielsen H, Brock JB (2008). Non-insulinoma persistent hyperinsulinaemic hypoglycaemia caused by an activating glucokinase mutation: hypoglycaemia unawareness and attacks. Clin Endocrinol.

[45] Christesen HT, Brusgaard K, Hussain K (2012). Recurrent spontaneous hypoglycaemia causes loss of neurogenic and neuroglycopaenic signs in infants with congenital hyperinsulinism. Clin Endocrinol.

[46] Ago Y, Sugie H, Fukuda T, Otsuka H, Sasai H, Nakama M (2019). A rare PHKA2 variant (p.G991A) identified in a patient with ketotic hypoglycemia. JIMD Rep.

[47] van Hasselt PM, Ferdinandusse S, Monroe GR, Ruiter JP, Turkenburg M, Geerlings MJ (2014). Monocarboxylate transporter 1 deficiency and ketone utilization. N Engl J Med.

[48] Al-Khawaga S, AlRayahi J, Khan F, Saraswathi S, Hasnah R, Haris B (2019). A SLC16A1 Mutation in an infant with ketoacidosis and neuroimaging assessment: expanding the clinical spectrum of MCT1 deficiency. Front Pediatr.

[49] Kishnani PS, Austin SL, Abdenur JE, Arn P, Bali DS, Boney A (2014). Diagnosis and management of glycogen storage disease type I: a practice guideline of the American College of Medical Genetics and Genomics. Genet Med.

[50] Eastman RC, Carson RE, Orloff DG, Cochran CS, Perdue JF, Rechler MM (1992). Glucose utilization in a patient with hepatoma and hypoglycemia: assessment by a positron emission tomography. J Clin Investig.

[51] Møller N, Schmitz O, Jørgensen JO, Astrup J, Bak JF, Christensen SE (1992). Basal- and insulin-stimulated substrate metabolism in patients with active acromegaly before and after adenomectomy. J Clin Endocrinol Metab.

[52] Shapiro ET, Bell GI, Polonsky KS, Rubenstein AH, Kew MC, Tager HS (1990). Tumor hypoglycemia: relationship to high molecular weight insulin-like growth factor-II. J Clin Investig.

[53] Santer R, Kinner M, Lassen CL, Schneppenheim R, Eggert P, Bald M (2003). Molecular analysis of the SGLT2 gene in patients with renal glucosuria. J Am Soc Nephrol.

[54] Koscielny G, Yaikhom G, Iyer V, Meehan TF, Morgan H, Atienza-Herrero J (2014). The International Mouse Phenotyping Consortium Web Portal, a unified point of access for knockout mice and related phenotyping data. Nucleic Acids Res.

[55] Marcus C, Alkén J, Eriksson J, Blom L, Gustafsson J (2007). Insufficient ketone body use is the cause of ketotic hypoglycemia in one of a pair of homozygotic twins. J Clin Endocrinol Metab.

[56] Beardsall K (2010). Measurement of glucose levels in the newborn. Early Hum Dev.

[57] Woo HC, Tolosa L, El-Metwally D, Viscardi RM (2014). Glucose monitoring in neonates: need for accurate and non-invasive methods. Arch Dis Child Fetal Neonatal Ed.

[58] Adamkin DH (2011). Postnatal glucose homeostasis in late-preterm and term infants. Pediatrics.

[59] Vanelli M, Mastrorilli C, Fainardi V, Iovane B, Scarabello C, Veronese P (2019). Clinical utility of beta-hydroxybutyrate measurement in the management of physiological ketosis at home in children under 5. Acta Biomed.

[60] Ross KM, Brown LM, Corrado MM, Chengsupanimit T, Curry LM, Ferrecchia IA (2016). Safety and efficacy of chronic extended release cornstarch therapy for glycogen storage disease type I. JIMD Rep.

[61] Currie G, Szabo J (2019). "It is like a jungle gym, and everything is under construction": the parent's perspective of caring for a child with a rare disease. Child Care Health Dev.

[62] Lopes MT, Koch VH, Sarrubbi-Junior V, Gallo PR, Carneiro-Sampaio M (2018). Difficulties in the diagnosis and treatment of rare diseases according to the perceptions of patients, relatives and health care professionals. Clinics (Sao Paulo).

